# Protected Mitral TEER With Temporary Ventricular Circulatory Support in Severe Left Ventricular Dysfunction for Heart Failure Management

**DOI:** 10.1016/j.jaccas.2026.108783

**Published:** 2026-06-24

**Authors:** Lukas Weber, Julien Ternacle, Johann Cattan, Melchior Jonveaux, Antoine Beurton, François Picard, François-Xavier Hérion, Thomas Modine, Lionel Leroux, Guillaume Bonnet

**Affiliations:** a Unité Médico-Chirurgicale des Valvulopathies Hôpital Cardiologique Haut-Lévêque, CHU de Bordeaux, Pessac, France; b Department of Cardiology, HOCH Health Ostschweiz, St Gallen, Switzerland; c Centre de Recherche Cardio-Thoracique de Bordeaux, University of Bordeaux, INSERM, Pessac, France; d Department of Cardiovascular Anesthesia and Critical Care, CHU Bordeaux, Bordeaux, France; e University of Bordeaux, INSERM, BMC, Pessac, France

**Keywords:** chronic heart failure, hemodynamics, mitral valve

## Abstract

**Background:**

Mitral transcatheter edge-to-edge repair (M-TEER) is an established therapy for secondary mitral regurgitation in heart failure, but its safety in patients with severely impaired left ventricular function is limited by the risk of afterload mismatch.

**Case Summary:**

We report a 46-year-old man with ischemic cardiomyopathy, left ventricular ejection fraction of 26%, and severe secondary mitral regurgitation who underwent planned M-TEER under elective Impella 5.0 (Abiomed) support via subclavian access. Two XTW clips were successfully implanted. The Impella was maintained postprocedure and weaned stepwise over several days, enabling gradual left ventricular adaptation, hemodynamic stability, and early rehabilitation.

**Discussion:**

Rapid reinitiation and uptitration of guideline-directed medical therapy led to sustained functional recovery and removal from the transplant list. At 3.5-year follow-up, the patient remains at NYHA functional class I-II status, with sustained clinical stability, improved exercise capacity, and no heart failure hospitalizations.

**Conclusions:**

This case illustrates a strategy combining elective M-TEER with temporary ventricular support to prevent afterload mismatch and safely reintroduce guideline-directed medical therapy in advanced heart failure.

## Visual Summary

**Figure:**
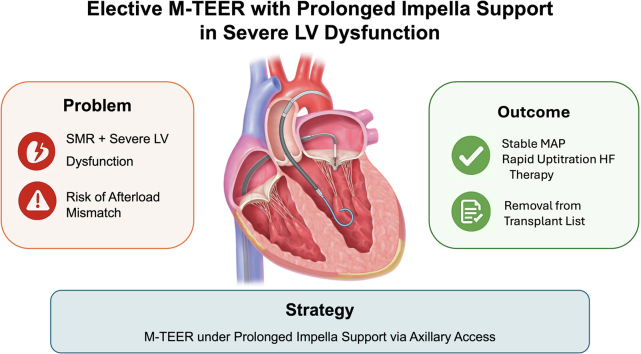

**Visual Summary:**
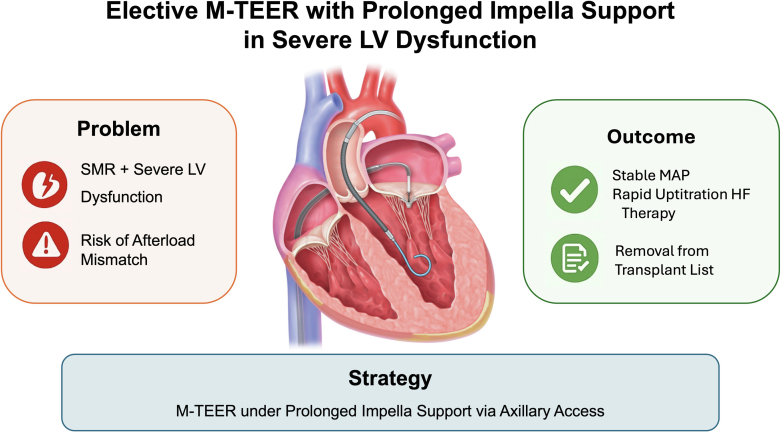
M-TEER Under Prolonged Impella Support in SMR and Severe LV Dysfunction to Mitigate Afterload Mismatch and Enable Hemodynamic Stabilization and Rapid Uptitration of Heart Failure Therapy HF = heart failure; LV = left ventricular; MAP = mean arterial pressure; M-TEER = mitral transcatheter edge-to-edge repair; SMR = secondary mitral regurgitation.

Mitral transcatheter edge-to-edge repair (M-TEER) is an established therapy for secondary mitral regurgitation (SMR) in heart failure (HF) patients with persistent symptoms despite optimal guideline-directed medical therapy (GDMT).^1^ The COAPT trial demonstrated improvements in symptoms, reduced hospitalizations, and lower mortality.^2^ However, COAPT excluded patients with severe left ventricular (LV) dilatation and/or systolic dysfunction—a population at high risk of afterload mismatch.^3^

**Take-Home Messages**

• Elective M-TEER with Impella support is feasible and safe in severe left ventricular dysfunction.
• Subclavian Impella enables patient mobilization and prolonged, stepwise weaning.
• Gradual left ventricular unloading prevents afterload mismatch and enables timely reinitiation of heart failure therapy.

In these patients, mitral regurgitation serves as a “low-impedance outlet” that unloads the failing LV by diverting blood flow into the low-pressure left atrium. Abrupt reduction in mitral regurgitation after M-TEER eliminates this LV unloading, potentially inducing acute afterload mismatch and precipitating LV failure. Implementing pre-emptive strategies to mitigate this risk is critical to improve outcomes in advanced HF candidates undergoing M-TEER.

Several reports have described M-TEER performed under mechanical hemodynamic support in the setting of cardiogenic shock^4^^,^^5^ and Muraca et al^6^ published 2 cases of planned “protected” TEER procedures with Impella support (Abiomed) that was withdrawn immediately postintervention.^6^ In contrast, we present a novel approach combining elective M-TEER with prolonged postprocedural temporary ventricular support, enabling stepwise LV adaptation, hemodynamic stabilization, early mobilization, and timely reintroduction of GDMT.

## History of Presentation

A 46-year-old patient with ischemic cardiomyopathy, after a prior anterior myocardial infarction treated with stenting of the left anterior descending artery and intermediate branch, was referred for the management of HF with reduced ejection fraction and severe SMR. Over the 10 months after his infarction, he experienced 3 HF hospitalizations and continued to report persistent exertional dyspnea (NYHA functional class III) despite optimal GDMT (Table 1).

**Table 1:** Initial Daily Doses of Heart Failure Guideline-Directed Medical Therapy

Medication	Daily Dose (mg)
Sacubitril/valsartan	97/103 twice a day
Bisoprolol	7.5
Eplerenone	50
Dapagliflozin	10

His only relevant comorbidity was a remote history of Hodgkin lymphoma, treated successfully with radiochemotherapy 2 decades earlier.

## Investigations

**Figure 1:**
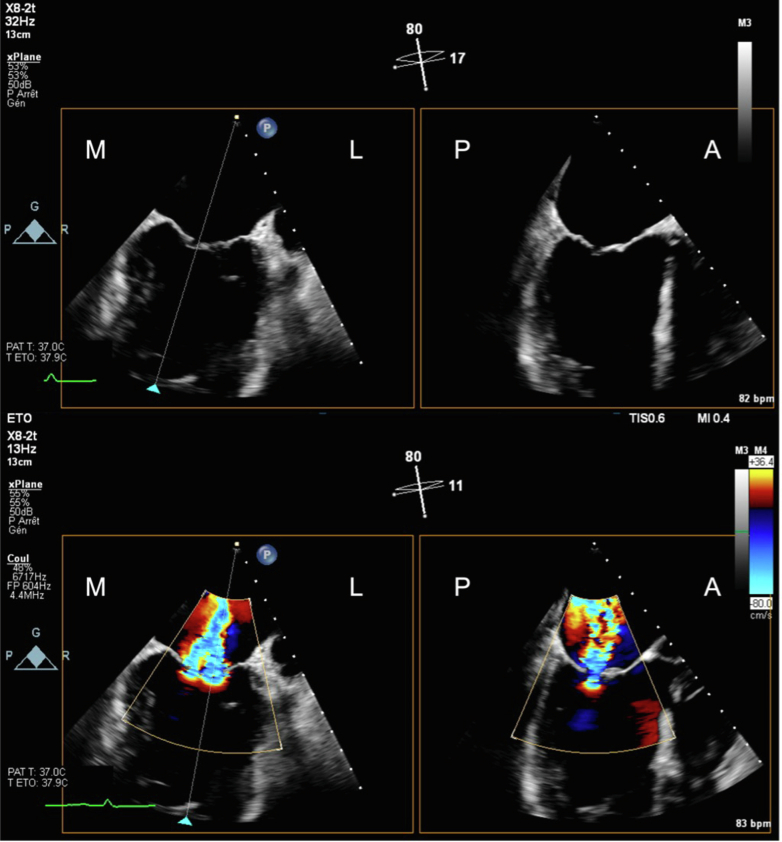
Preprocedural Transesophageal Echocardiographic Findings A = anterior; L = lateral; M = medial; P = posterior.

On ambulatory evaluation systolic blood pressure was 93 mm Hg without overt signs of congestion. The electrocardiogram showed sinus rhythm with a narrow QRS complex. N-terminal pro–B-type natriuretic peptide was markedly elevated (3,879 pg/mL), and renal function was moderately impaired (estimated glomerular filtration rate: 48 mL/min/m^2^). Key baseline clinical characteristics and laboratory findings are summarized in Table 2. Transthoracic and transesophageal echocardiography (TEE) revealed a severely dilated LV and reduced systolic function (left ventricular ejection fraction [LVEF]: 26%). Severe ventricular SMR was observed, driven by marked leaflet tethering producing 2 central jets, yet without a relevant coaptation gap (Figure 1, Videos 1 to 3). The transmitral mean gradient was 2 mm Hg. There were no valvular or annular calcifications, and the posterior leaflet measured 15 mm, supporting the feasibility of TEER. There was no additional hemodynamically significant valvular disease. The key baseline transthoracic echocardiography parameters are summarized in Table 3.

**Table 2:** Key Baseline Clinical Characteristics and Laboratory Findings

Age (y)	48
Sex	Male
Height (cm)	178
Weight (kg)	83
Body mass index (kg/m^2^)	26.2
NYHA functional class	III
Heart rate (beats/min)	84
Blood pressure (mm Hg)	93/68
Heart rhythm	Sinus rhythm
STS risk score	2.3
Hemoglobin (g/dL)	13.5
Platelets (G/L)	273
Creatinine (μmol/L)	145
eGFR (mL/min/m^2^)	48
Potassium (mmol/L)	5.1
NT-proBNP (pg/mL)	3,879

eGFR = estimated glomerular filtration rate; NT-proBNP = N-terminal pro–B-type natriuretic peptide; STS = Society of Thoracic Surgeons.

**Table 3:** Key Baseline Transthoracic Echocardiography Parameters

LV end-diastolic dimension	6.5 cm
LV end-diastolic volume	210 mL
LV ejection fraction	26%
Stroke volume	27 mL
RV FAC	32%
Effective regurgitant orifice area	0.5 cm^2^
Regurgitant volume	62 mL
Mean transmitral gradient	2 mm Hg
Mitral valve area (3D)	6.5 cm^2^
Anterior leaflet length	26 mm
Posterior leaflet length	15 mm
Tethering height	12 mm
Estimated sPAP	58 mm Hg

3D = 3-dimensional; LV = left ventricular; RV FAC = right ventricular fractional area change; sPAP = systolic pulmonary artery pressure.

Cardiac magnetic resonance imaging demonstrated transmural necrosis of the mid anteroseptal and inferoseptal segments involving the entire apical crown and the inferobasal segment, consistent with severely impaired LV systolic function (LVEF: 18%). Cardiopulmonary exercise testing revealed severely reduced functional capacity (Vo_2_ max: 17 mL/kg/min). Right heart catheterization (Table 4) demonstrated severe postcapillary pulmonary hypertension with elevated filling pressures (mean pulmonary arterial pressure: 43 mm Hg, mean pulmonary arterial wedge pressure: 33 mm Hg) and a large V-wave up to 49 mm Hg, underscoring the hemodynamic burden of mitral regurgitation.

**Table 4:** Baseline Right Heart Catheterization Data

PAP (systolic/diastolic/mean)	61/28/43 mm Hg
PCWP (mean)	33 mm Hg
V wave	49 mm Hg
Right atrial pressure	10 mm Hg
Cardiac output	5.1 L/min
Cardiac index	2.5 L/min/m^2^
PVR	2 WU
Svo_2_	69%

PAP = pulmonary artery pressure; PCWP = pulmonary capillary wedge pressure; PVR = pulmonary vascular resistance; Svo_2_ = mixed venous oxygen saturation.

In this context, severe SMR was considered the main driver of clinical deterioration, and the patient was considered for advanced HF therapies, including potential heart transplantation or durable LV assist device implantation.

## Management

After a multidisciplinary heart team discussion, the patient was scheduled for elective M-TEER using the MitraClip system (Abbott), under mechanical circulatory support with a coaxial-flow pump (Impella 5.0, Abiomed) to prevent afterload mismatch during the days after mitral regurgitation reduction. The Impella device was favored over an intra-aortic balloon pump given the need for prolonged circulatory support via subclavian access, thereby enabling early mobilization—an approach not feasible with femoral access. Concurrent listing for potential heart transplantation was maintained in case of clinical deterioration.

Hospital admission was scheduled 2 days before the intervention for meticulous preparation, including volume optimization and tailored adjustment of HF pharmacotherapy. Angiotensin receptor–neprilysin inhibitors and mineralocorticoid receptor antagonists were temporarily discontinued. From a physiologic perspective, Impella implantation 48 hours before intervention, in synchrony with the suspension of part of the HF therapy, would have been ideal. However, this strategy was not feasible because of logistic constraints.

**Figure 2:**
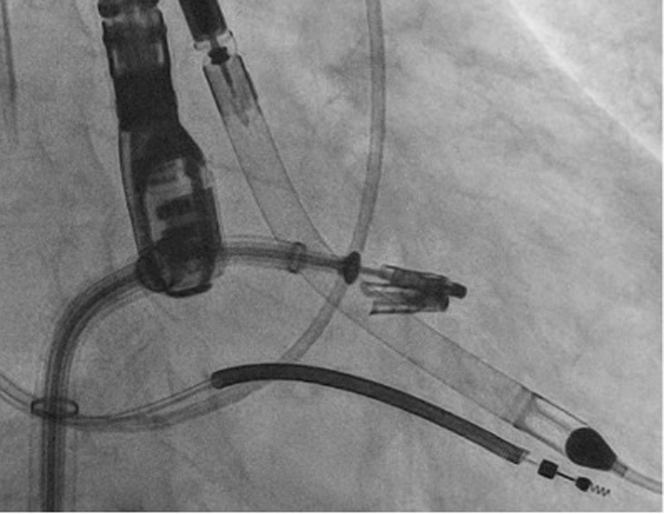
Fluoroscopic Image Showing the Deployment of the Second XTW Clip and the Inserted Impella 5.0 on Right Anterior Oblique View

**Figure 3:**
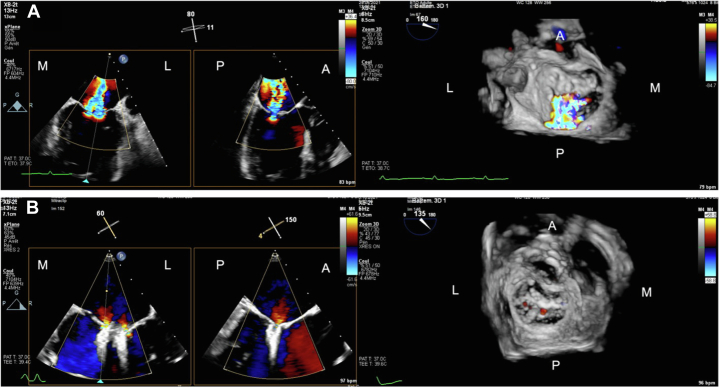
Preprocedural and Postprocedural Transesophageal Echocardiography (A) Preprocedural and (B) postprocedural transesophageal echocardiography showing the optimal result with residual trace regurgitation. A = anterior; L = lateral; M = medial; P = posterior.

Under general anesthesia, the Impella 5.0 was surgically inserted via the left subclavian artery (which subsequently enabled early mobilization with the device in place) and positioned across the aortic valve. Guided by TEE, 2 XTW Clips were deployed in A2/P2 segments (Figure 2). The presence of the active Impella device did not impede the technical performance of the MitraClip deployment, nor did it significantly affect TEE image quality. The postprocedural TEE demonstrated an optimal result with residual trace mitral regurgitation and a mean transmitral gradient of 5 mm Hg (Figure 3).

**Figure 4:**
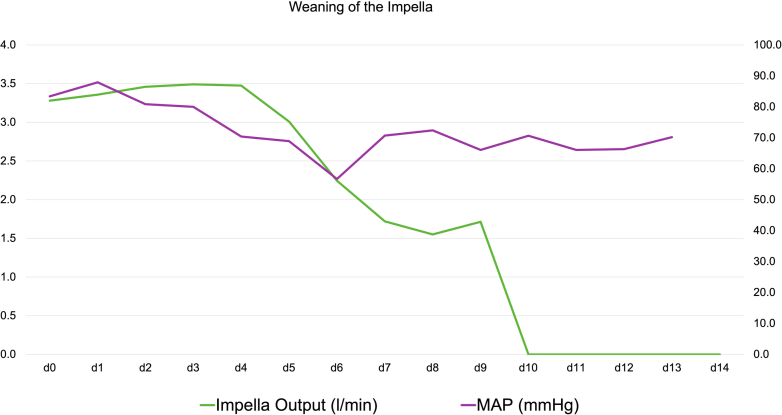
Weaning of Impella Support with Stable Mean Arterial Pressure MAP = mean arterial pressure.

**Figure 5:**
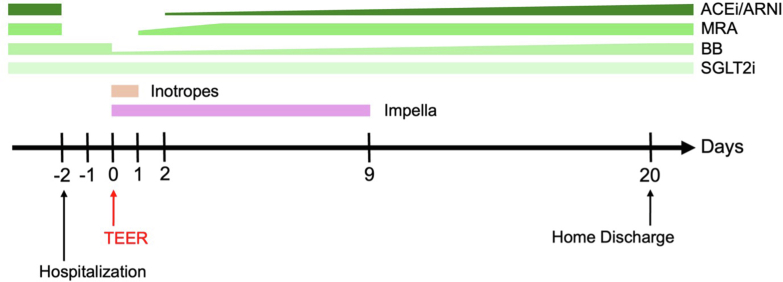
Timeline With Postprocedural Weaning of the Impella and Retitration of Heart Failure Medication ACEi/ARNI = angiotensin converting enzyme inhibitor/angiotensin receptor–neprilysin inhibitor; BB = beta-blocker; MRA = mineralocorticoid receptor antagonist; SGLT2i = sodium-glucose cotransporter 2 inhibitor; TEER = transcatheter edge-to-edge repair.

The postoperative course was uneventful. The patient was extubated early, required no oxygen therapy, and was mobilized promptly. The Impella was gradually weaned over several days without the need for prolonged inotropic support, while mean arterial pressure (MAP) remained stable (Figure 4). HF pharmacotherapy was reinitiated and progressively uptitrated following a stepwise strategy illustrated schematically in Figure 5, with detailed daily dosing provided in Supplemental Table 1. Impella management was primarily guided by MAP, with a target MAP of ≥60 mm Hg. Daily hemodynamic parameters during the Impella support and stepwise weaning period are summarized in Supplemental Table 2. In addition, serum lactate levels were monitored regularly and remained within the normal range throughout the weaning period, indicating adequate systemic perfusion. The Impella was removed 9 days after the procedure, and the patient was discharged on day 20 with the maximum tolerated established 4-pillar HF regimen, including maximal dose of angiotensin receptor–neprilysin inhibitor and mineralocorticoid receptor antagonist.

## Outcome and Follow-Up

At 1 month, the patient exhibited a clear symptomatic relief associated with substantial improvement in objective prognostic markers of heart failure. N-terminal pro–B-type natriuretic peptide had fallen to 1,200 pg/mL, peak Vo_2_ increased to 22 mL/kg/min, and pulmonary pressures had nearly normalized (mean pulmonary arterial pressure: 23 mm Hg, mean pulmonary arterial wedge pressure: 18 mm Hg).

These favorable changes enabled the patient's removal from the heart transplantation list. Over a follow-up period now extending to 3.5 years, he has remained paucisymptomatic (NYHA functional class I-II) and clinically stable, without recurrent HF hospitalizations.

## Discussion

This case demonstrates the feasibility, safety, and potential benefits of performing planned M-TEER using Impella mechanical circulatory support in patients with severe SMR and severely impaired LV systolic function. Although current guidelines do not advocate the use of advanced LV support devices for percutaneous structural interventions,^1^ this report suggests that the use of such devices could be considered in carefully selected high-risk patients.

Most prior reports of M-TEER with mechanical support have focused on patients in cardiogenic shock or acute mitral regurgitation. Muraca et al^6^ described 2 “protected” elective procedures with Impella support, but in both instances, assistance was withdrawn immediately or within hours after the intervention.^6^ The novelty of our approach lies in extending Impella support for several days after M-TEER, allowing the ventricle to gradually adapt to the otherwise abrupt increase in afterload. Conceptually, this strategy resembles a stepwise closure of the MitraClips over several days, progressively eliminating the “low-impedance outlet.” This approach not only reduces the risk of afterload mismatch—a well-recognized driver of periprocedural instability and mortality^7^, ^8^, ^9^, ^10^—but also avoids the need for catecholamines, such as dobutamine, which may exacerbate myocardial oxygen demand and provoke adverse effects including arrhythmias or, in rare cases, leaflet tear.

Moreover, maintaining temporary LV unloading after M-TEER provides a protected hemodynamic environment for the early reintroduction and uptitration of GDMT, a crucial component of long-term disease modification that is often challenging to implement in this population because of hypotension and poor perfusion.

## Conclusions

This case describes the use of combined M-TEER and prolonged Impella support in advanced HF patients with severe SMR. By LV support over several days with gradually weaning, the ventricle is given time to adapt, minimizing hemodynamic instability and facilitating rapid optimization of HF therapy. This may represent a potential management approach for complex, stable, high-risk HF cases and warrants further evaluation.

## Funding Support and Author Disclosures

This case report received funding through a research grant provided by Abiomed. Dr Ternacle is a consultant for Abbott Structural and Edwards Lifesciences. Drs Modine and Leroux and are consultants for Abbott Structural, Medtronic, and Edwards Lifesciences. Dr Bonnet is a consultant for Medtronic. All other authors have reported that they have no relationships relevant to the contents of this paper to disclose.

## Appendix

Video 1 TEE X-Plane View Before M-TEER

Video 2 TEE X-Plane View With Color Doppler Before M-TEER

Video 3 Three-Dimensional TEE Surgical View Before M-TEER
