# Supplementary material for: Protected Mitral TEER With Temporary Ventricular Circulatory Support in Severe Left Ventricular Dysfunction for Heart Failure Management
Source: JACC Case Rep. 2026 Jun 24;31(25):108783. doi: 10.1016/j.jaccas.2026.108783 (PMC13332643; doi:10.1016/j.jaccas.2026.108783)
Supplement: Supplemental Table 1 and 2 [file mmc4.docx]

**SUPPLEMENTARY MATERIAL**

**Supplementary Table 1:** Day-by-day titration of heart failure therapies (in mg per molecule, twice a day for sacubitril/valsartan) during the intensive care unit stay, with final medical therapy at hospital discharge (Day 20).

|  | **d0** | **d1** | **d2** | **d3** | **d4** | **d5** | **d6** | **d7** | **d8** | **d9** | **d10** | **d11** | **d20** |  |
| --- | --- | --- | --- | --- | --- | --- | --- | --- | --- | --- | --- | --- | --- | --- |
| Sacubitril/Valsartan | - | - | 24/26 | 24/26 | 24/26 | 24/26 | - | - | - | - | - | - | 97/103 |  |
| Ramipril | - | - | - | - | - | - | - | 2.5 | 1.25 | 2.5 | 1.25 | 1.25 | - |  |
| Bisoprolol | 2.5 | 2.5 | 2.5 | 2.5 | 2.5 | 2.5 | 2.5 | 2.5 | 2.5 | 2.5 | 2.5 | 2.5 | 5 |  |
| Eplerenone | 25 | 50 | 50 | 50 | 50 | 50 | 50 | 50 | 50 | 50 | 50 | 50 | 50 |  |
| Dapagliflozin | 10 | 10 | 10 | 10 | 10 | 10 | 10 | 10 | 10 | 10 | 10 | 10 | 10 |  |

**Supplementary Table 2:** Daily hemodynamic parameters during Impella weaning. HR = heart rate, SAP = systolic arterial pressure, DAP = diastolic arterial pressure, MAP = mean arterial pressure, SPAP = systolic pulmonary arterial pressure, SVO2 = mixed venous oxygen saturation, CI = Cardiac index.

|  | **d0** | **d1** | **d2** | **d3** | **d4** | **d5** | **d6** | **d7** | **d8** | **d9** | **d10** | **d11** |
| --- | --- | --- | --- | --- | --- | --- | --- | --- | --- | --- | --- | --- |
| **Mean HR (bpm)** | 79 | 90 | 92 | 82 | 83 | 84 | 89 | 89 | 86 | 85 | 95 | 84 |
| **Mean SAP (mmHg)** | 112 | 111 | 105 | 92 | 89 | 87 | 76 | 83 | 88 | 86 | 92 | 86 |
| **Mean DAP (mmHg)** | 72 | 73 | 69 | 64 | 60 | 59 | 44 | 58 | 59 | 55 | 60 | 57 |
| **Mean MAP (mmHg)** | 83 | 88 | 81 | 80 | 70 | 69 | 57 | 71 | 73 | 66 | 71 | 66 |
| **Mean SPAP (mmHg)** | 38 | 39 | 41 | 30 | 27 | 29 | 32 | 27 | 28 | 36 | 24 | 14 |
| **Mean SVO2 (%)** | 82 | 76 | 69 | 70 | 69 | 66 | 69 | 66 | 66 | 76 | 78 | 75 |
| **Mean CI (l/min/m2)** | 4.0 | 3.7 | 4.1 | 3.2 | 3.3 | 3.8 | 3.2 | 2.9 | 2.5 | 3.4 | 3.4 | 3.1 |
